# Improved multilineage human hematopoietic reconstitution and function in NSGS mice

**DOI:** 10.1371/journal.pone.0209034

**Published:** 2018-12-12

**Authors:** Mark Wunderlich, Fu-Sheng Chou, Christina Sexton, Pietro Presicce, Claire A. Chougnet, Julio Aliberti, James C. Mulloy

**Affiliations:** 1 Division of Experimental Hematology and Cancer Biology, Cancer and Blood Disease Institute, Cincinnati Children’s Hospital Medical Center, Cincinnati, Ohio, United States of America; 2 Division of Immunobiology, Cincinnati Children’s Hospital Medical Center, Cincinnati, Ohio, United States of America; University of California, San Francisco, UNITED STATES

## Abstract

Genetic manipulation of NOD/SCID (NS) mice has yielded numerous sub-strains with specific traits useful for the study of human hematopoietic xenografts, each with unique characteristics. Here, we have compared the engraftment and output of umbilical cord blood (UCB) CD34+ cells in four immune-deficient strains: NS, NS with additional IL2RG knockout (NSG), NS with transgenic expression of human myeloid promoting cytokines SCF, GM-CSF, and IL-3 (NSS), and NS with both IL2RG knockout and transgenic cytokine expression (NSGS). Overall engraftment of human hematopoietic cells was highest in the IL2RG knockout strains (NSG and NSGS), while myeloid cell output was notably enhanced in the two strains with transgenic cytokine expression (NSS and NSGS). In further comparisons of NSG and NSGS mice, several additional differences were noted. NSGS mice were found to have a more rapid reconstitution of T cells, improved B cell differentiation, increased levels of NK cells, reduced platelets, and reduced maintenance of primitive CD34+ cells in the bone marrow. NSGS were superior hosts for secondary engraftment and both strains were equally suitable for experiments of graft versus host disease. Increased levels of human cytokines as well as human IgG and IgM were detected in the serum of humanized NSGS mice. Furthermore, immunization of humanized NSGS mice provided evidence of a functional response to repeated antigen exposure, implying a more complete hematopoietic graft was generated in these mice. These results highlight the important role that myeloid cells and myeloid-supportive cytokines play in the formation of a more functional xenograft immune system in humanized mice.

## Introduction

Immunodeficient mice have been used to study human hematopoiesis for decades. The advent of the NOD/SCID (NS) mouse was a key development that greatly improved the consistency and ease of xenograft studies. However, this strain is hampered by several traits limiting its use, including susceptibility to endogenous spontaneous lymphomas beginning as soon as 5–6 months of age [1]. Residual innate immune function from NK cells limits engraftment of human hematopoietic stem cells [2] [3]. Furthermore, established grafts decline over time, are markedly biased to the B cell lineage, develop only a minimal myeloid component [4], and do not develop any NK or T cells [5].

Numerous attempts to modify the NS mouse have been made in an effort to improve human xenografts. Currently the most successful strain modifications have centered on genetic inactivation of interleukin-2 receptor gamma (IL2RG). Two such strains exist, one with expression of a truncated IL2RG lacking the cytoplasmic domain (NOG) [6] and a second with a full gene deletion (NSG) [7, 8]. In both cases, these mice have a further reduced innate immunity as a result of diminished macrophage and NK activity. As a result, these mice are highly immune-compromised and significantly more sensitive to lethal infection by common infectious agents [9]. However, the total block in lymphoid development also suppresses endogenous lymphoma development and results in a much longer lifespan, given proper husbandry techniques. Studies of long-term hematopoiesis that were not possible can now be performed in the xenograft setting. Both NSG and NOG are capable of supporting robust, long-term, B cell dominated grafts that over time include significant T and NK cell populations [6, 10]. In light of these advances, NSG and NOG mice are currently the most frequently employed strains for xenograft studies of normal human hematopoiesis. While these two strains are highly similar, it has been proposed that the extracellular portion of IL2RG may retain some limited function and allow signaling to a minimal degree by way of hetero-dimerization with a subset of its target receptor complexes. Indeed, one study has found a slight advantage for NSG over NOG mice in their role as hosts for CD34+ cells, particularly at low cell doses of CD34+ cells [11].

While NOG and NSG mice solve several NS problems, these mice still have grafts that consist mainly of lymphoid cells. Study of human myeloid biology remains challenging. The decreased myeloid compartment likely affects the functionality and comprehensiveness of the graft as a whole, particularly when innate immunity or antigen presentation is necessary. Additionally, the lack of myeloid cells might result in a lack of important cytokine signals that cannot be supplied by the mouse environment. In order to address this shortfall, the NOD/SCID-SGM3 (NSS) mouse was developed that constitutively expresses the human myelo-supportive cytokines SCF, GM-CSF, and IL-3 (SGM3) [12]. While it was shown that the NSS mouse promotes myeloid cell development from fetal liver (FL) or bone marrow (BM) CD34+ cells, relatively little has been done to characterize these mice using standard xenograft approaches.

Several sources of HSCs are available, each with unique characteristics. Human BM, UCB, and FL HSCs have each been used for generation of humanized mice. FL CD34+ purified cells appear to engraft NSG mice more efficiently than UCB CD34+ cells [13, 14]. This may be due to higher levels of primitive HSCs and multi-potent progenitors and fewer B progenitors in FL relative to UCB [14]. FL xenografts have been reported to display more immature B cells and smaller follicular structures compared to UCB engrafted mice, while BM HSCs generate comparatively poor levels of engraftment with reduced functionality [15].

We crossed the NSG (IL2RG knockout) with the NSS (SGM3 expression) strains to obtain a mouse with all the advantages of the NSG with an increased myeloid potential that could promote better AML xenografts, the NSGS mouse. We showed that NSGS mice produced more robust grafts than NSG mice when AML cell lines as well as AML primary patient BM samples were injected into matched cohorts [16]. Some patient samples that failed to engraft NSG mice yielded significant grafts in NSGS mice, demonstrating a strict dependence of a subset of these samples on myeloid cytokine signals and revealing an increased sensitivity for human AML leukemic initiating cells, consistent with the previous finding that exogenous cytokines can aid CD34+ engraftment when low cell numbers are infused [17]. Similar results have also recently been reported for MDS, CMML, and JMML [18–20], indicating a benefit of SGM3 expression for a broad range of myeloid neoplasms.

Others have also studied NSGS mice and evaluated the hematopoietic output of xenografts. Human FL CD34+ cells produced increased myeloid cells as well as increased Tregs that were capable of inhibiting T proliferation [21]. A bone marrow-fetal liver-thymus (BLT) model using NSGS mice showed improved maturation of B cells which were capable of production of antigen specific IgG antibodies [22]. Increased myeloid output from short term repopulating cells [23] and long term increased myeloid cell development [22, 24] have also been described. Here, we have engrafted NS, NSS, NSG, and NSGS mice with UCB CD34+ cells and evaluated the reconstitution and functionality of the resulting xenografts.

## Materials and methods

### Ethics statements

Human UCB tissue was procurred by the Translational Trials Development Support Laboratories of CCHMC with the approval of an institutional review board approved protocol (#02-3-4x) which includes informed consent of the donors.

The mouse work in this study was approved by the Institutional Animal Care and Use Committee of Cincinnati Children’s Hospital Medical Center (protocol#2017–0080). Mice were humanely euthanized by carbon dioxide exposure followed by cervical dislocation.

### Mice

NOD.CB17-Prkdc^scid^/J (NS, The Jackson Laboratory #001303), NOD.Cg-Prkdc^scid^Il2rg^tm1Wjl^/SzJ (NSG, The Jackson Laboratory #005557), NOD.Cg-Prkdc^scid^Tg(CMV-IL3,CSF2,KITLG) (NSS, kindly provided by Dr. Connie Eaves, [12]), and NOD.Cg-Prkdc^scid^ Il2rg^tmWjl^Tg(CMV-IL3,CSF2,KITLG)1Eav/MloySzJ (NSGS, The Jackson Laboratory #013062 [16]) were bred in a pathogen free facility at the Cincinnati Children’s Hospital Research Foundation according to standard practices under an approved protocol.

### CD34+ xenografts

**Fig 1:**
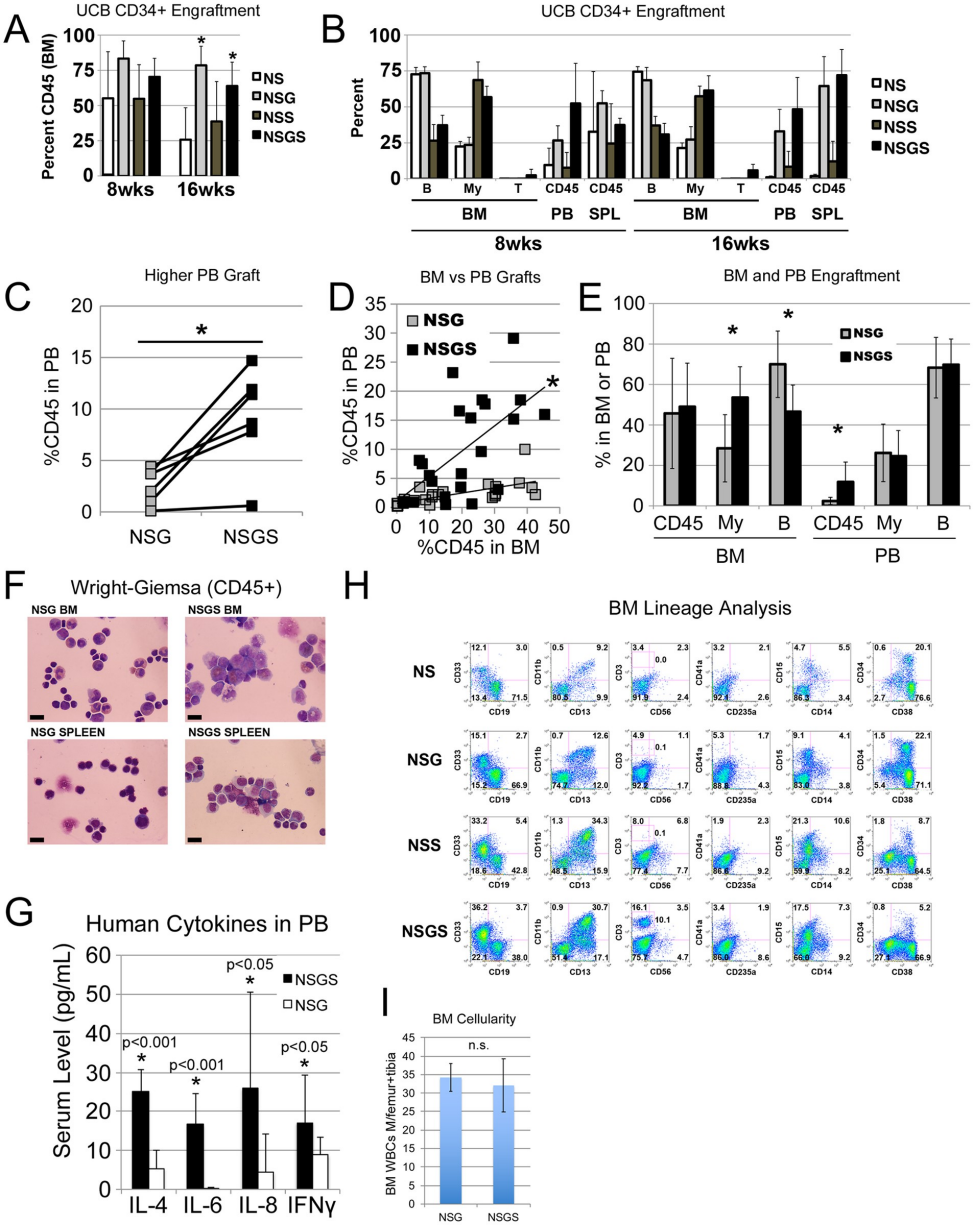
Four strain comparison of engraftment. A) Total human engraftment (CD45+) in the BM of mice at 8 and 16 weeks. B) Lineage composition of the human grafts from the BM, PB and spleen. B cells (CD19+), myeloid cells (CD13+ and CD33+), and T cells (CD3+) were assessed within the CD45+ population. C) PB CD45+ levels in mice engrafted with 6 unique UCB CD34+ preparations 6–8 weeks after engraftment. D) PB grafts from mice in “C” were plotted against the BM grafts from the same mice. The slopes of the trendlines were judged to be different by linear regression analysis, Prism 7 software, p = 0.0023. E) Lineage analysis of BM and PB for the grafts in “C”. F) Wright Giemsa stained cytospins of FACS sorted CD45+ cells from BM and spleen. G) Human cytokine serum concentrations in humanized mice. H) Flow cytometric analysis of BM grafts at 16 weeks from some mice included in experiments presented in panels A and B. I) Average total BM WBCs in 1 femur and 1 tibia (combined) were determined for NSG and NSGS mice at 15 weeks of engraftment. Total cellularity was not significantly different. Asterisks indicate p value less than 0.05 by Student’s T Test.

Umbilical cord blood (UCB) units were collected by the Translational Trials Development Support Laboratory of Cincinnati Children’s Hospital Research Foundation and distributed according to an approved IRB protocol. The mononuclear cell fraction was obtained after density separation through a ficoll gradient (Ficoll Paque Plus, GE Healthcare). CD34+ cells were selected with the EasySep human CD34+ magnetic isolation kit (StemCell Technologies). For initial experiments (Fig 1), approximately 150,000 cells from UCB CD34+ preparations of >95% purity were used for intrafemoral injections of 6–8 week old immunodeficient mice conditioned 4–6 hours prior with sub-lethal irradiation (280 Rad) from a cesium-137 (gamma ray) source. Subsequently, all CD34+ cells were delivered intravenously at a dose of 50,000 cells per mouse. Mice received doxycycline chow (0.0625%, Purina) previous to and up to 2 weeks post irradiation and transplantation as a prophylactic measure against possible irradiation-induced systemic infections. For some experiments, UCB was CD34+ enriched to approximately 60% before they were further FACS sorted to acquire highly pure CD34+CD3- cells. These T cell negative preparations were used in similar experiments at a dose of 50,000 cells per mouse. BM aspirations and tail vein bleeds were performed to monitor the grafts according to standard protocols [25].

### Xenogenic GVHLD

Age-matched mice conditioned with or without 200 Rad of irradiation received intravenous injections of 10 million normal human donor peripheral blood mononuclear cells (PBMNCs). Samples were obtained with an IRB approved protocol and proper consent. Mice were monitored for signs of xenogenic graft versus host disease (GVHD) including severe weight loss and expansion of human T cells circulating in the peripheral blood.

### Flow cytometry

Flow cytometry was performed on FACSCanto instruments (BD Bioscience) and data analysis was performed with FlowJo software (TreeStar, Inc). Antibody information is included as a supplemental table (S1 Table). Sorted hCD45+ BM and spleen samples were obtained from a FACSAria instrument (BD).

### Immunization

*Toxoplasma gondii* extract (STAg) was prepared from laboratory strains of T gondii. Mice were given two or three separate intraperitoneal STAg injections spaced 3 days apart. For DTH experiments, a final exposure to STAg was given by footpad injection. Footpad swelling was measured 24 hours later with calipers and normalized to the change in thickness observed in the PBS injected contralateral foot. Serum was obtained from these mice in order to perform an ELISA to detect Toxoplasma specific antibodies. Spleen cells were harvested and cultured in the presence or absence of STAg followed by ELISA detection of human IFNγ in order to demonstrate recall.

## Results

### Human hematopoiesis is significantly altered in SGM3+ immunodeficient mice

To characterize the effect of human SGM3 cytokines on xenografts, we tested 3 independent UCB CD34+ preparations simultaneously in NS, NSG, NSS (NS with SGM3), and NSGS (NSG with SGM3) mice. Saturating doses of UCB CD34+ cells were injected directly into the BM cavity of irradiated mice to maximize the grafts. At 8 weeks post-transplant, we consistently observed high levels of human cell engraftment in all mice. However, while grafts in NS and NSS mice dropped noticeably at 16 weeks, those of NSG and NSGS mice remained high and were significantly higher than NS (Fig 1A). Higher percentages of human grafts were detected in the peripheral blood (PB) and spleen of NSG and NSGS mice, particularly at the later time point (Fig 1B). These results confirm other findings that mice with IL2RG knockout show superior and more durable human grafts [7]. Additionally, under these conditions, transgenic SGM3 expression in mice did not significantly diminish the durability of the graft during the 16-week observation period, despite the previous findings describing loss of primitive cells and LTC-IC activity in NSS mice [12].

In these initial experiments, we noted a slightly higher level of human cells in the PB of NSGS mice relative to matched NSG mice (Fig 1B). This finding was subsequently confirmed by measuring human cells in the PB of NSG and NSGS mice 6–8 weeks after intravenous engraftment of CD34+ cells from an additional 6 UCB samples (Fig 1C). To determine whether this effect was due to a greater propensity of the NSGS graft to peripheralize, we analyzed BM and PB grafts in the same mice. When plotted together, individual PB/BM grafts revealed distinctly different patterns in these two strains, with the NSGS cohort displaying a significantly steeper slope indicating greater peripheralization of the graft (Fig 1D).

**Fig 2:**
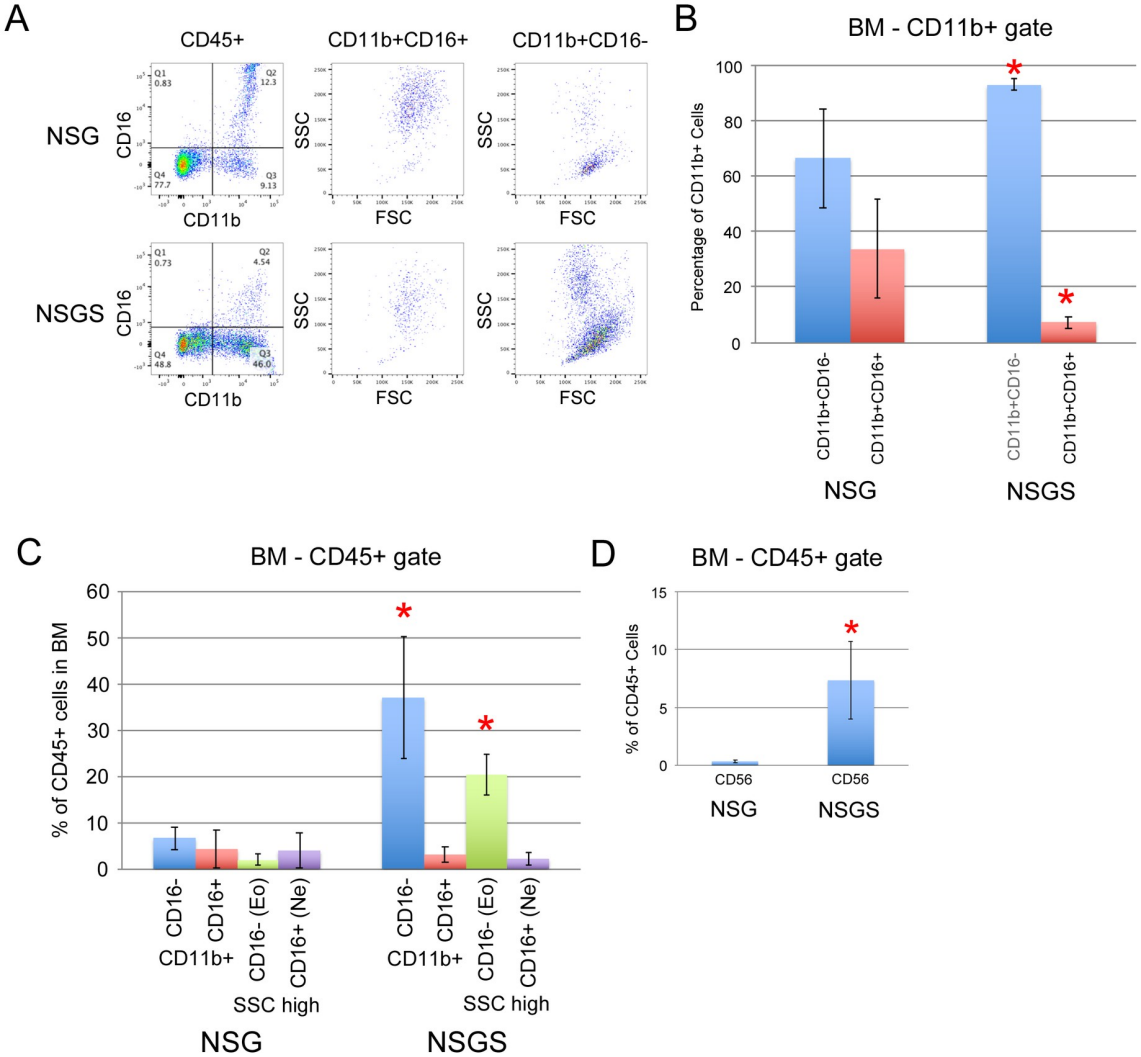
Differential myeloid and NK differentiation in NSGS compared to NSG. A) Representative FACS analysis of BM from UCB CD34+ engrafted mice co-stained with CD11b and CD16 antibodies at 15 weeks of engraftment. Average and standard deviation of a cohort of mice stained and analyzed as in A and represented as percentage of B) CD11b+ cells or C) CD45+ cells. D) Average percentage of CD56+ NK cells in BM of the same engrafted mice. Asterisks indicate p value less than 0.05 by Student’s T Test.

**Fig 3:**
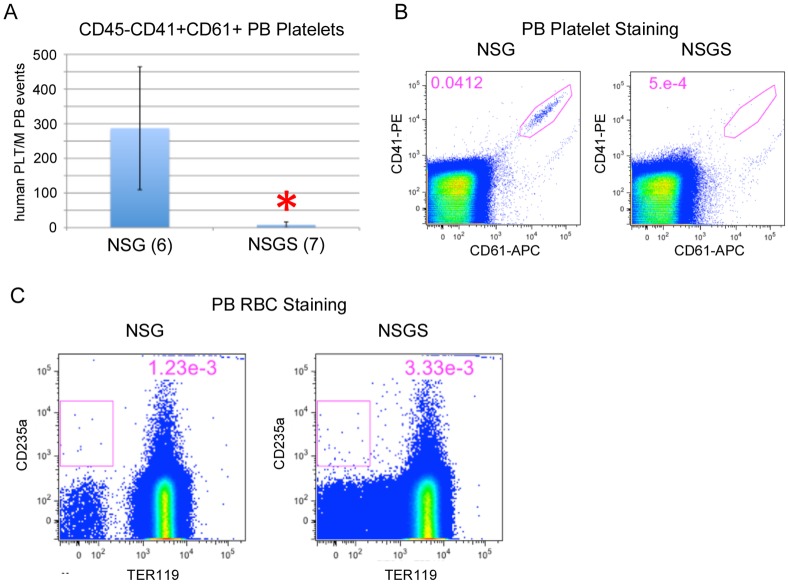
Reduced platelets and lack of RBCs in NSGS PB. A) PB from UCB-engrafted mice was stained for platelet markers CD41 and CD61. Shown are average and standard deviation. B) Representative FACS plots. C) Whole blood staining demonstrating a lack of human CD235a RBCs in both NSG and NSGS PB. Mice were engrafted for 15 weeks at the time of data collection. Asterisks indicate p value less than 0.05 by Student’s T Test.

**Fig 4:**
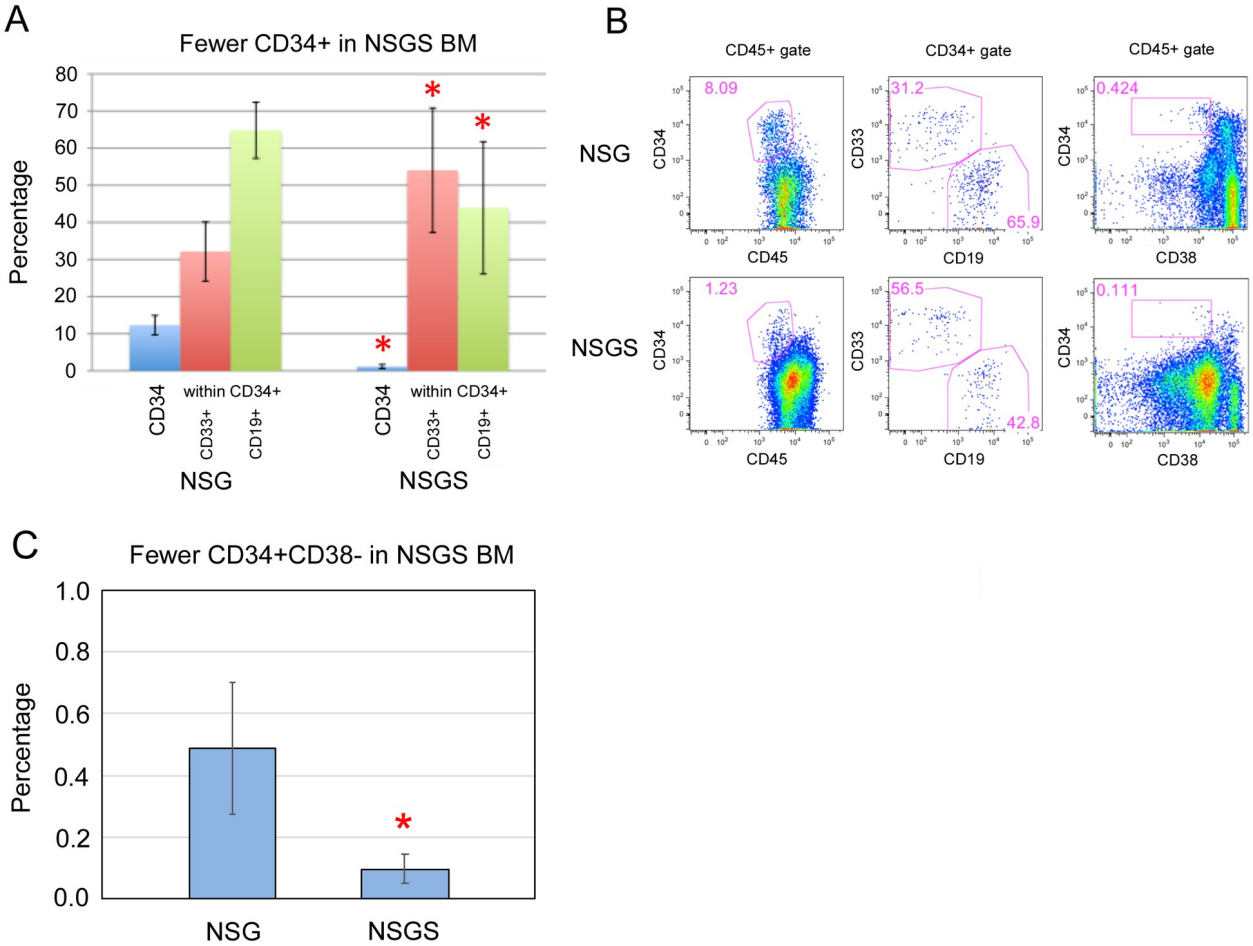
Reduced CD34+ cells in BM are myeloid skewed. A) Averages and B) representative FACS analysis of UCB-engrafted BM demonstrating higher CD33+ and lower CD19+ co-staining on CD34+ cells from NSGS mice engrafted for 15 weeks. C) Primitive CD34+CD38- cells were also significantly reduced in NSGS BM (flow example also shown in B). Asterisks indicate p value less than 0.05 by Student’s T Test.

We analyzed the lineage composition of the grafts and found significant increases in CD13/CD33+ myeloid cells and a concomitant decrease in CD19+ B cells in the BM of NSGS and NSS mice. Interestingly, this bias was not observed in the PB (Fig 1B and 1E). Additionally, we sorted hCD45+/mCD45- cells from the BM and spleen of engrafted mice and examined cell morphology by Wright-Giemsa staining. A higher frequency of large cells with monocytic appearance were detected in NSGS BM and spleen, while most human cells in NSG mice resembled lymphocytes among scattered myeloid cells (Fig 1F). Consistent with these findings, we observed elevated levels of human IL-4, IL-6, and IL-8 in the sera of humanized NSGS mice, all cytokines that are produced by macrophage cells (Fig 1G). Flow cytometric analysis of BM aspirates from engrafted mice confirmed the shift to CD33+ myeloid cells and decrease in CD19+ B cells in both SGM3+ strains (NSS and NSGS) (Fig 1H). Importantly, total cellularity in the BM was not significantly different in UCB transplanted NSG and NSGS mice at 15 weeks of engraftment, therefore percentage engraftment is reflective of the total relative absolute cell number of the populations observed (Fig 1I). Consistent with previous findings, we reproducibly detected small populations of CD3+ T cells in the BM of NSGS mice [21]. Generally, grafts in all four strains contained similar subpopulations of myeloid marked cells (CD11b, CD13, CD14, and CD15) despite the obvious differences in relative number of these cells. Notably, myeloid cells were more likely to be CD11b in the SGM3+ mice, implying some potential qualitative differences in myeloid differentiation. Further examination showed a differential pattern of CD16 expression on CD11b positive cells consistent with neutrophil differentiation (CD16^high^, SSC^high^) in NSG mice and eosinophil (CD16-, SSC^high^) and monocyte differentiation (CD16-, FSC^high^, SSC^low^) in NSGS mice (Fig 2A–2C). Other notable differences in the SGM3+ mice were an increased level of CD56+CD3- NK cells to levels similar to those described in a recent study [26] (Figs 1H and 2D) and nearly complete absence of circulating platelets, which were readily detectable in NSG (Fig 3A and 3B). Circulating RBCs were not detected in the PB of either strain (Fig 3C). A reduced population of both CD34+ progenitor cells, which were noted to be more skewed towards CD33+ myeloid commitment, as well as more primitive CD34+CD38- cells was observed in NSGS (Fig 4A and 4B).

### NSGS mice do not retain SCID-repopulating cell (SRC) activity but are more sensitive to SRC engraftment

**Fig 5:**
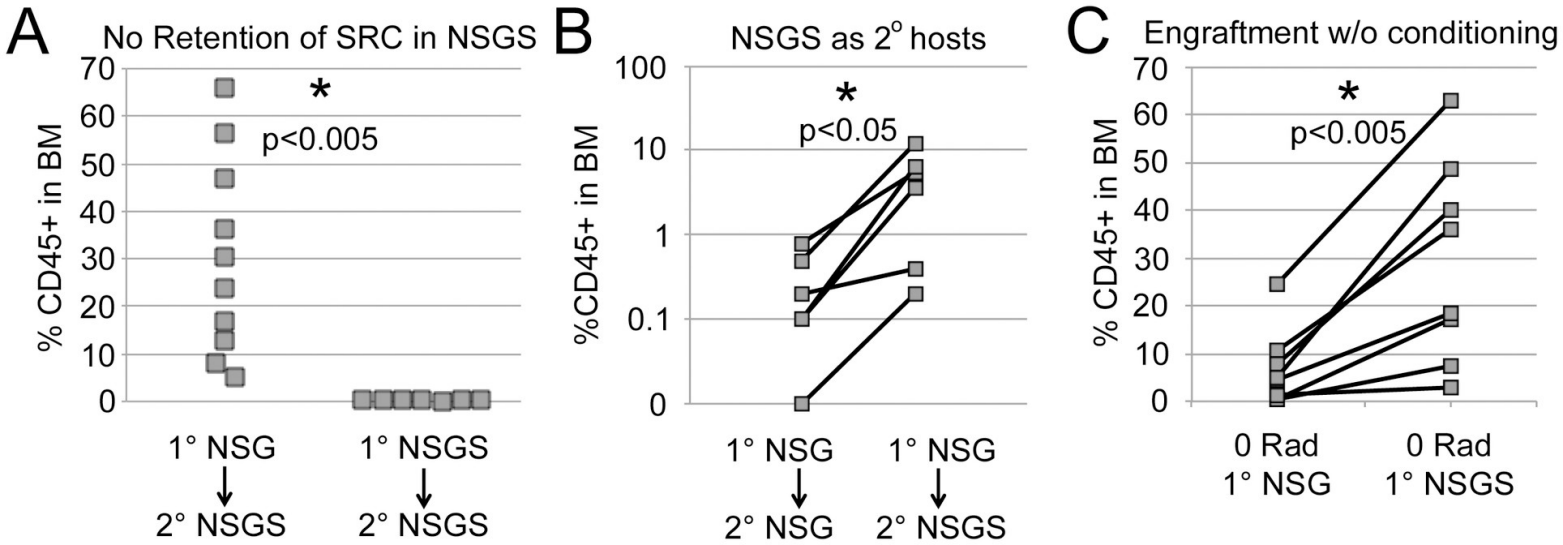
NSGS have increased sensitivity to SRCs. A) Whole BM from primary engrafted NSG and NSGS mice (15 weeks) was used for secondary transplant into NSGS mice. Only NSG BM had detectable SRC activity at 8 weeks after transplant. B) BM from primary NSG mice (15 weeks) was used for paired secondary transplant into NSG and NSGS mice. The NSGS mice engrafted significantly better at 8 weeks. C) A series of UCB CD34+ preparations were engrafted into non-conditioned NSG and NSGS and BM engraftment was measured at 10 weeks. Asterisks indicate p value less than 0.05 by Student’s T Tests.

The lower level of CD34+ cells suggested that the human SGM3 cytokines drive myelopoiesis at the expense of primitive stem and progenitor cell preservation, as previously suggested [12]. To measure SRC activity of the human grafts in NSGS mice, secondary transplants were performed using BM from a cohort of NSG and NSGS mice engrafted with the same UCB CD34+ sample (Fig 5A). BM from NSG and NSGS xenografted mice were transferred to conditioned NSGS secondary recipients. The grafts from primary NSGS mice failed to establish significant secondary grafts, indicating a paucity of SRCs in the primary mice. In contrast, NSG BM samples consistently yielded robust secondary BM engraftment. In fact, these results were much better than would be expected from our experience with NSG to NSG serial transplants, suggesting that NSGS may be a preferred secondary host. To test this possibility, we compared secondary engraftment potential of grafts from primary NSG mice into age- and sex-matched secondary mice. Secondary grafts were significantly higher in NSGS mice (Fig 5B).

As an additional stringent test for sensitivity to SRCs, we tested engraftment of NSG and NSGS mice without conditioning. A previous report showed that such engraftment was feasible in NSG mice engrafted with UCB-CD34+ cells by intrafemoral injection [27]. Despite the fact that we used intravenous delivery, we were also able to detect multi-lineage engraftment using five unique UCB CD34+ preps in the majority of our recipients. We observed a significantly increased engraftment in NSGS mice. Relative to NSG, there was an average 4.6-fold higher graft in PB and a 4.3-fold increase in BM of NSGS recipients (Fig 5C). While the exact nature of the cells that engraft non-conditioned hosts remains to be characterized, these data support the conclusion that NSGS have increased sensitivity to SRCs.

### Rapid T cell development in NSGS mice

**Fig 6:**
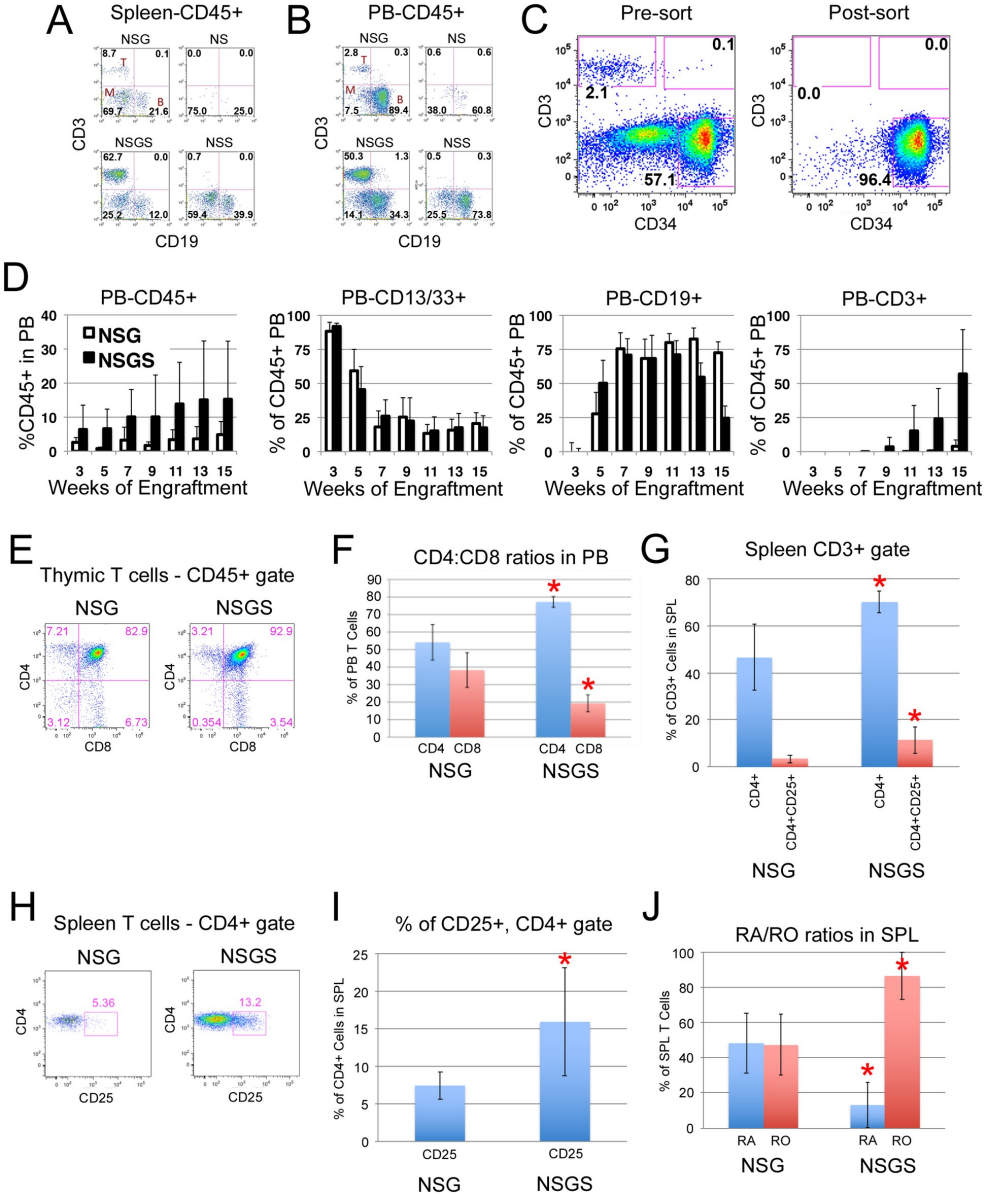
Rapid T cell differentiation in NSGS mice. Representative flow plots from week 16 engrafted mice showing lineage distribution in A) spleen and B) PB. C) Flow plots showing potential T cell contamination in CD34+ isolations that was removed by FACS sorting CD34+CD3- cells. D) Kinetics of total human, B, myeloid, and T cells engraftment in the PB of NSG and NSGS mice engrafted with the same FACS sorted UCB CD34+CD3- samples. E) Thymus FACS analysis shows normal double positive primitive T cells. F) PB CD4 and CD8 frequencies among CD3+ T cells. G) Spleen Tregs were enumerated. A representative flow plot example is shown in H and the data is displayed as percentage of CD4 cells in I. J) RA/RO ratios in spleen CD3+ cells. Panels E-J were completed 15 weeks after engraftment. Asterisks indicate p value less than 0.05 by Student’s T Test.

During our initial experiments with NSGS mice, we consistently noticed the early appearance of CD3+ human T cells in NSGS mice, as also reported previously (Fig 1)[21]. Additionally, at 16 weeks, these T cells were more abundant in NSGS spleen (Fig 6A) and PB (Fig 6B) than was detected in age-matched NSG mice engrafted in parallel. To further explore this finding and to rule out expansion of contaminating T cells from the UCB CD34+ cell preparations in NSGS mice, we undertook additional experiments using CD34+CD3- sorted samples injected into NSG and NSGS mice (Fig 6C). PB was monitored for human (CD45+, Fig 6D left), myeloid (CD13/CD33+, left center), B (CD19+, right center), and T (CD3+, right) cells every two weeks beginning at week 3. In both NSG and NSGS mice the PB grafts were characterized by a predominance of myeloid cells that eventually reached a low steady-state level by 6–7 weeks. In contrast, the B cell component in PB was negligible until week 7 when it quickly increased to dominate the graft. The PB graft composition did not qualitatively differ between NSG and NSGS until sometime between weeks 7–9 when T cells first appeared in some NSGS mice. Notably, T cell development correlated with a decreased percentage of B cells while myeloid cell contribution to the graft remained steady. These data are in sharp contrast to the BM and spleen composition of NSG and NSGS grafts, indicating a significant difference in homeostasis depending on the hematopoietic organ sampled (Fig 1B). Consistent with previous studies, we first detected T cell production in NSG mice beginning around 14 weeks, a delay of 4–6 weeks relative to NSGS grafts. Taken together these data suggest that the human T cells in NSGS grafts are derived from primitive cells in vivo and are not an artifact of T cell contamination of the CD34+ preparations.

Next, we sought to compare the T cells subsets found in NSG and NSGS mice. Thymus preparations from long-term engrafted mice were found to contain a majority of human T cells at the CD4+CD8+ stage typical of primitive T cell differentiation (Fig 6E). T cells in circulation were significantly skewed to the CD4 lineage in NSGS grafts, with a higher percentage of these co-staining for CD25 expression, indicating higher Treg differentiation as previously reported (Fig 6F–6I) [21]. There was also a notable increase in staining for CD45RO (memory T) at the expense of CD45RA (naïve T) in T cells isolated from NSGS spleens (Fig 6J).

### Similar rate of expansion of differentiated T cells in NSG and NSGS mice

**Fig 7:**
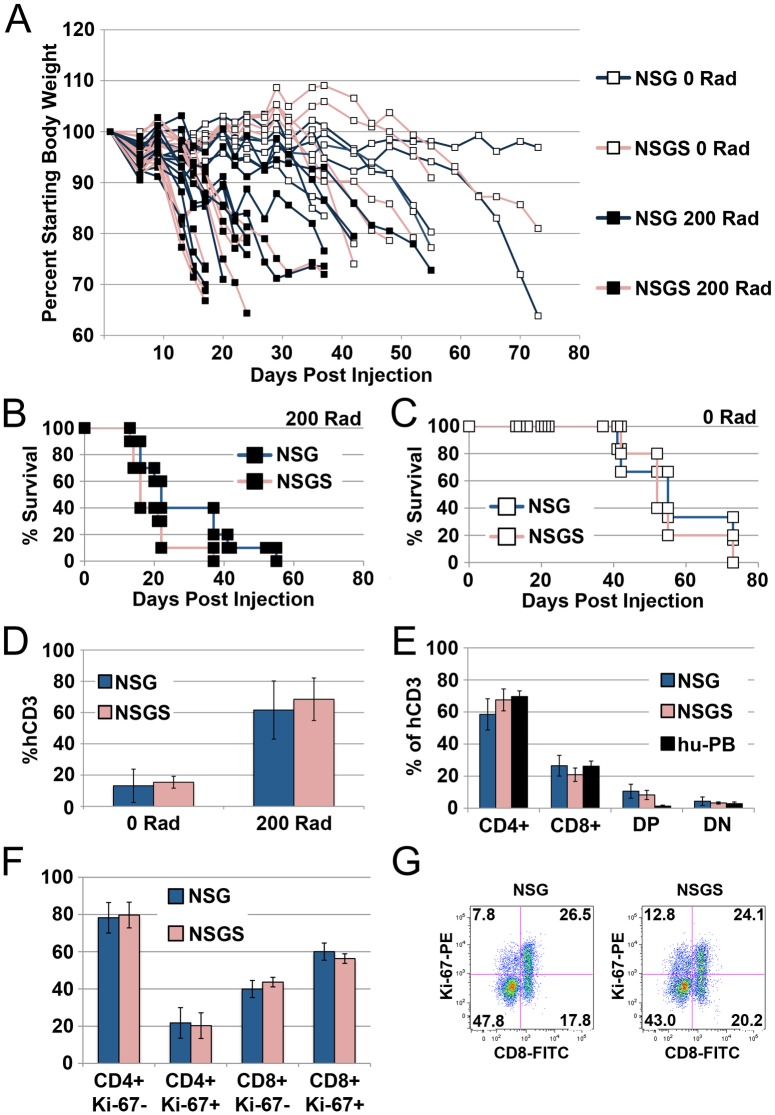
Mature T cells expand similarly in NSG and NSGS as shown by GVHD models. A) Individual mouse weights over time after tail vein injection of adult PB MNCs. NSG (blue lines) and NSGS (red lines) experience weight loss with similar kinetics. Conditioned mice (filled boxes) show weight loss before non-conditioned mice (open boxes). B-C) Survival of the mice in panel A. D) Levels of CD3+ T cells in mice at 2 weeks. E) Levels of CD4 and CD8 cells at 2 weeks. F) Ki-67 staining reveals no changes in the cycling of either CD4 or CD8 T cells between strains. G) Representative plot used to generate panel F.

NSG and NOG mice have been used as hosts for xenogeneic graft versus host disease (GVHD) [28–30]. We made use of these models in order to examine the nature of T cell expansion in matched NSG and NSGS mice injected with normal donor PB mononuclear cells. We sought to determine whether or not enhanced expansion of differentiated human T cells could explain the high T cell levels in NSGS mice. We observed no difference in the induction of GVHD in NSG and NSGS mice preconditioned with a 200 Rad irradiation dose as measured by weight loss (Fig 7A) or mortality (Fig 7B). Similar results were also noted when we used non-conditioned mice (Fig 7C). This approach has been shown to delay the onset of GVHD [28] and therefore we might expect greater sensitivity for detecting differences due to lower initial T cell engraftment. However, in both models using both approaches, PB human T cell frequencies were very similar during GVHD development (Fig 7D). Furthermore, the CD4/CD8 ratio and cell cycle status of the T cells did not show any differences (Fig 7E–7G). These data support the notion that the T cell phenotype observed in humanized NSGS mice results from improved T cell commitment, thymus seeding, or enhanced differentiation from primitive cells and not expansion or survival of T cells once they appear.

### NSGS spleens contain human cells arranged in follicle like structures

**Fig 8:**
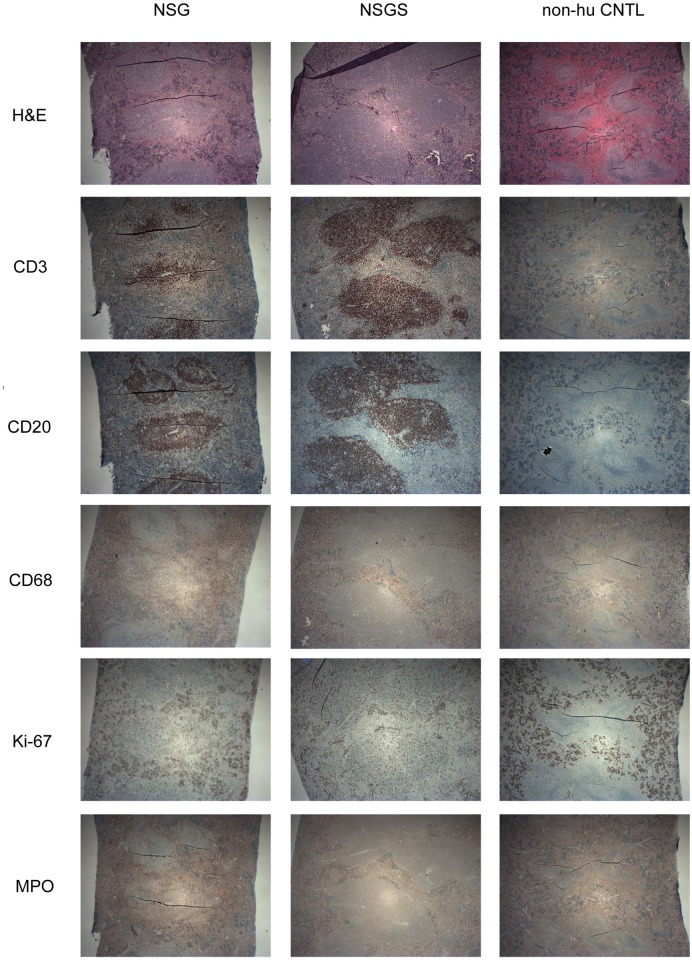
Humanized NSGS mice exhibit proper splenic organization. H&E or the indicated antibody stains were performed on formalin fixed spleen tissue from long term engrafted mice (16 weeks).

To characterize the spatial arrangement of the human graft in the spleen, serial sections were made and stained using human specific antibodies. Staining highlighted several densely packed areas consistent with white pulp within the broader red pulp of the spleen (Fig 8). These areas showed intense staining for both human B (CD20) and T (CD3) cells. Macrophage cells (CD68) are also evident throughout the spleen in both strains. MPO staining was darker in engrafted NSG which may correspond to more granulocytic myeloid differentiation [31]. Many cells of both human and murine origin were positive for Ki-67, signifying proliferation. Together these data indicate that the human graft is successfully arranging into perivascular compartments in the mouse spleen, mimicking normal patterns seen in human tissue.

### Humanized NSGS produce human IgG and IgM antibodies

**Fig 9:**
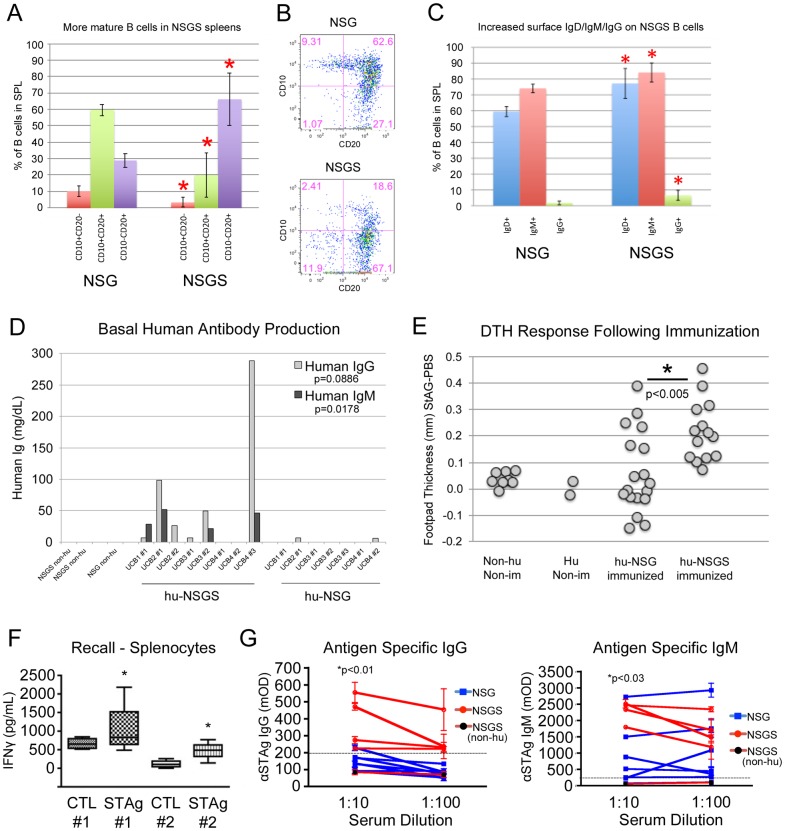
Humanized NSGS mice have more functional immune systems than humanized NSG mice. A) FACS analysis showing the average population distribution for the indicated marker combinations at 15 weeks of engraftment. B) Representative flow plots. C) IgD/IgM/IgG surface expression was measured on B cells from spleens at 15 weeks. D) Serum from long term engrafted mice (15–16 weeks) was tested for the presence of human IgG and IgM. E) Mice immunized three times with a toxoplasma extract were challenged with a final injection of extract or PBS into the footpads. Swelling was measured 24 hours later and thickness of the PBS-injected footpad was subtracted from the thickness of the extract-injected footpad. F) Splenic cell isolates from these mice were subjected to STAg extract or PBS in vitro and the media was tested for release of IFNy. G) Serum from the mice was tested for toxoplasma specific IgG and IgM antibodies by ELISA assays. Asterisks indicate p value less than 0.05 by Student’s T Test or paired T Test (panel D, F).

We examined the differentiation state of B cells isolated from humanized spleens. Human B cells from NSGS mice were more likely to be CD10-CD20+, demonstrating more complete differentiation and development. (Fig 9A and 9B). They also displayed slightly increased surface expression of IgD, IgM and IgG (Fig 9C). Naïve mouse serum was tested for human IgG, IgM, and IgA. Consistent with previous reports, we observed very little serum antibody in hu-NSG mice. In contrast, we readily detected both human IgG and IgM antibodies in most hu-NSGS serum samples (Fig 9D). This finding strongly implies that the hematopoietic grafts are capable of adaptive immune responses.

### Hu-NSGS mice mount an immune response to repeated antigen exposure

The improved graft development, composition, structural organization and human cytokine production in NSGS mice compelled us to test the functionality of the human immune graft as a whole. Mice were immunized by injection of STAg, a crude extract of the intracellular parasite *Toxoplasma gondii*. As a test of immune response, we performed delayed-type hypersensitivity (DTH) experiments in which STAg was injected into the footpad of previously immunized mice. 24-hour Footpad swelling at 24 hours was significant in humanized NSGS mice while very slight or no effects were detected in humanized NSG or non-humanized controls (Fig 9E). Spleen preparations taken from immunized mice exhibited significant recall activity in vitro as measured by IFNγ release in response to STAg exposure (Fig 9F). Additionally, Toxoplasma-specific human IgG and IgM antibodies were more consistently detected in humanized NSGS mice compared to humanized NSG mice which generally gave signals more similar to non-humanized control mouse serum, especially for IgG (Fig 9G). Together, these results suggest that the unique human grafts found in NSGS are capable of recognition of Toxoplasma antigens and mount an immune response to those antigens.

## Discussion

In this study, we show that NSGS mice display all the benefits of IL2RG knockout as well as a significantly increased ability to promote myeloid growth and differentiation in the BM and spleen of humanized mice. The NSGS strain serves as a more sensitive readout for the measurement of rare SCID repopulating cells (SRCs). The early appearance of human T cells in the peripheral blood indicated a quickened development of a multi-lineage immune graft, with increased human cytokine concentrations as well as human IgG and IgM antibodies detected in the serum of humanized NSGS mice. Immunization against *Toxoplasma gondii* led to a significant DTH response and the generation of antigen specific IgG in hu-NSGS mice, indicating improved graft functionality in these mice.

We consistently observed significantly improved engraftment in non-conditioned NSGS hosts relative to NSG mice. This finding may be explained by the chronic transgenic SCF expression in NSGS mice. SCF has been shown to enhance the mobilization of HSCs by G-CSF [32]. Additionally, it was shown recently that immunodeficient mice with loss-of-function mutant kit alleles possess more open niches and allow for robust engraftment of human HSCs without conditioning [33]. NSG mice expressing membrane bound human SCF also supported higher graft levels from human HSCs [34]. Together, these studies show that dysregulated c-kit/SCF signaling often results in enhanced HSC engraftment.

NSG mice have significantly improved serial transfer of hematopoietic grafts relative to that seen in historic immunodeficient mice, but the model still likely underestimates the frequencies of normal and leukemic HSC and LIC present in human samples [35]. More sensitive models would improve studies that aim to identify single cells that possess stem cell activity [36]. Administration of exogenous cytokines to NS mice has been used to boost engraftment from samples with limited SRC numbers but the level of improvement was inadequate [17]. As expected from reports of the NSS stain, we found that human cytokine expression in NSGS significantly reduced maintenance of primitive human progenitors and SRCs in the BM of engrafted mice, resulting in lack of serial transfer ability of NSGS grafts. However, NSGS served as a far superior transplant recipient from primary NSG mice, promoting multi-lineage grafts that were significantly better than parallel NSG recipients. This ability will enable more sensitive measurement of SRCs in experiments that depend on demonstration of self-renewal of human cells in vivo. The follow up finding that adult non-conditioned NSGS mice can be used as hosts for UCB CD34+ engraftment confirms the significantly improved sensitivity of these mice for SRCs.

NSGS mice had higher proportions of CD4+ helper T, memory CD45RO+ T, Tregs, B cells that were more differentiated, and more abundant monocyte/macrophages that could serve as potential APCs. This mix of differences in graft quality relative to NSG mice resulted in a humanized mouse more capable of mounting an immune response. The exact mechanism and the particular changes most responsible for this improvement remain unknown. It is possible that the higher IL-4 levels, possibly produced by the increased Th2 T cells, and/or the higher IL-10 levels found in humanized NSGS mice [37] are inducing human B cell class switching and result in a more functional immune response. The UCB-NSGS humanized mouse offers a relatively straight forward model to examine such interplay of human immune cell signaling in vivo.

*Toxoplasma gondii* is an intracellular protozoan parasite with promiscuous infectivity towards birds and mammals. While the infection is often lifelong in the brain and muscle tissues of the host it invades, in man the result is typically a chronic benign infection resulting from effective suppression. Studies in mice indicate that entering this maintenance phase is dependent on efficient early cytokine signals including IFNy that activate innate immune function during the initial acute infection phase [38]. The innate immunity, particularly macrophage and dendritic cells, are known to actively combat infection during these early timepoints. Long term control of the parasite in a benign state seems to rely on proper adaptive immunity from B and T cells. A xenograft model of *Toxoplasma gondii* infection would be useful to determine the effects on and response from human immune cells, especially early during the initial response because there is no opportunity to study this in patients. In fact, the specific Toll-like receptors and the immunity-related GTPases that mice have evolved to deal with T. gondii infection are not present in the human genome [39]. Previous attempts at a xenograft model that focused on infusion of human peripheral blood leukocytes have resulted in little immune response [40, 41].

Several other models have recently been published that show similar immune responses in humanized mice. NSG mice induced to express human IL-3, IL-4, IL-7, IL-15, GM-CSF, and M-CSF by adeno-associated virus transfer and then engrafted with fetal liver CD34+ cells mounted an immune response that included respiratory syncytial virus specific IgG and IgA production and resulted in lower viral burden in the lungs [42]. The same system has been used to demonstrate similar results after immunization and challenge with the malaria causing parasite, *P*. *berghei* [43]. Notably, the levels of GM-CSF and IL-3 were very similar in that system [44] as are found in the NSGS mouse [16].

Several groups have engineered MHC class I or II molecules into NSG mice through transgenic expression of specific human alleles. These models, in particular those with class II molecule expression, produce impressive functional responses when engrafted with matched normal donor stem cells [45–49]. It will be interesting to couple transgenic MHC expression with the NSGS mouse.

## Supporting information

S1 Table Antibody list. (XLSX)
